# Cerebral embolic protection (CEP) during transcatheter aortic valve replacement (TAVR) is associated with a lower rate of stroke at a high-volume center

**DOI:** 10.1007/s00392-025-02802-5

**Published:** 2025-11-24

**Authors:** Michael Paukovitsch, Julia Lorenz, Dominik Felbel, Jinny Scheffler, Marvin Krohn-Grimberghe, Leonhard Moritz Schneider, Johannes Mörike, Wolfgang Rottbauer, Dominik Buckert

**Affiliations:** Department of Cardiology, Ulm University Heart Center, Albert-Einstein-Allee 23, 89081 Ulm, Germany

**Keywords:** Transcatheter aortic valve replacement, Stroke, Cerebral embolic protection

## Abstract

**Background:**

Cerebral embolic protection (CEP) reduces strokes during transcatheter aortic valve replacement (TAVR) but is not standard of care at most centers.

**Aim:**

To assess the impact of CEP use in real-world practice at a tertiary center using CEP as a standard of care during TAVR.

**Methods:**

In-hospital outcome of 2173 patients was compared to 328 (13.1%) patients who could not receive CEP during TAVR due to anatomical or technical reasons. In a secondary analysis, adjusted stroke risk was compared using propensity score matching.

**Results:**

Non-CEP patients had significantly higher Society of Thoracic Surgeons score for mortality (5.2 [interquartile range (IQR): 3.3–7.9] vs. 3.4% [IQR: 2.2–5.9], *p* < 0.01) and were more often female (54.0 vs. 46.4%, *p* = 0.01). Comorbidities such as coronary artery disease (63.4 vs. 61.7%, *p* = 0.54) and prior cardiac surgery (11.9 vs. 9.6%, *p* = 0.19) were equally frequent in both groups, whereas a history of prior stroke (16.2 vs. 11.7%, *p* = 0.02) was more frequent in non-CEP patients. Despite significantly longer procedure time in CEP patients (55.0 min [IQR: 46.0–66.0] vs. 53.0 min [43.0–63.3], *p* < 0.01), intraprocedural death (0 vs. 0.1%, *p* = 1.0), arrhythmia (11.9 vs. 11.9%, *p* = 0.99), and vascular access-site complications (5.5 vs. 4.3%, *p* = 0.32) were equally frequent. Although intraprocedural stroke occurred seldomly in both patient groups (0 vs. 0.3%, *p* = 1.0), in-hospital disabling stroke occurred more often in non-CEP patients (4.0 vs. 1.8%, *p* = 0.01). In the propensity score matched cohort, CEP use was associated with a significantly lower risk of all stroke (OR: 0.41, 95% CI: 0.22–0.77, *p* < 0.01) as well as disabling stroke (OR: 0.37, 95% CI: 0.18–0.78, *p* < 0.01).

**Conclusion:**

At a high-volume center using CEP as part of its standard of care during TAVR, CEP use was associated with a lower rate of in-hospital stroke. Especially those patients who could not receive CEP seemed to be at increased risk for stroke.

**Graphical Abstract:**

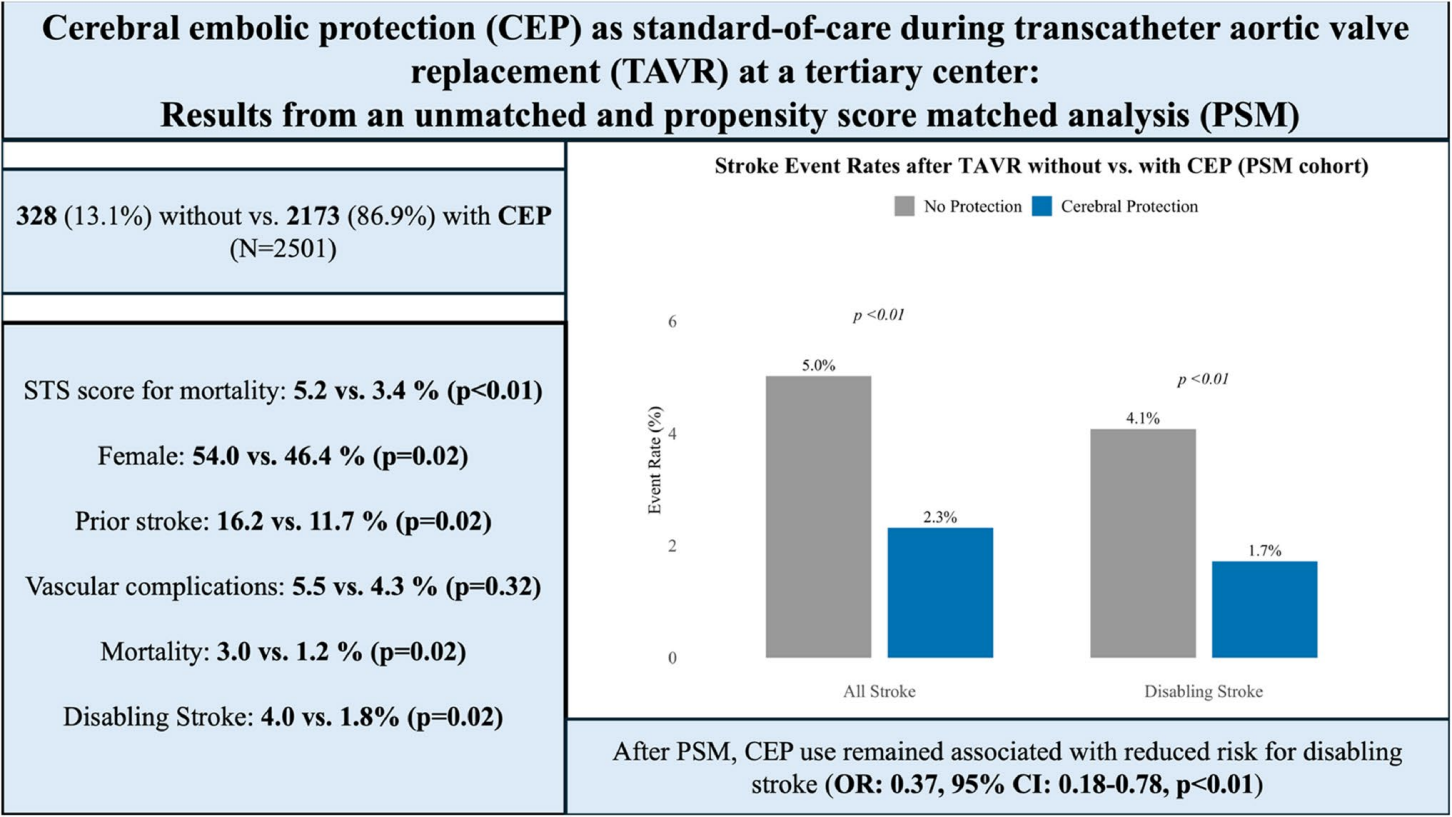

Two thousand five hundred one patients were analyzed according to CEP use. CEP could be used in the majority of patients, whereas a minority (13.1%) of patients could not receive CEP for technical or anatomical reasons. Non-CEP patients had significantly higher procedural risk and experienced in-hospital stroke more often.

**Supplementary Information:**

The online version contains supplementary material available at 10.1007/s00392-025-02802-5.

## Introduction

Ischemic stroke occurs in 2–3% [1–3] of patients undergoing transcatheter aortic valve replacement (TAVR) and is associated with increased mortality [2–5]. Although the safety profile of TAVR has improved regarding mortality, vascular complications, and acute kidney injury [6], a large, multicenter pooled patient-level study including over twenty thousand patients reported a stable incidence of stroke over recent years [1]. Another study pooling data from North American centers made a similar observation [3]. The majority of strokes occur within a week after TAVR [7] peaking within the first 24 h [2, 7, 8] after the procedure.

Hence, stroke remains one of the most frequent and dreaded complications [6]. Cerebral embolic protection (CEP) devices, which are inserted during a TAVR procedure and removed upon completion, have been shown to reduce cerebral lesions on dedicated cerebral imaging [9, 10]. Furthermore, such cerebral lesions after TAVR may negatively impact cognitive function [11]. Observational and propensity-score matched studies found CEP to be associated with a reduction of clinically evident strokes [12, 13]. However, CEP use could not show a significant reduction of in-hospital stroke in the multicenter randomized-controlled PROTECTED TAVR trial using the Sentinel device [8]. A trend towards a reduction of disabling strokes was observed in that trial and the authors concluded that a benefit of CEP could not be ultimately ruled out [8].

In addition to the Sentinel device, further CEP devices, which either function as filters or deflectors of circulating debris, have been introduced or are under investigation [14, 15]. Despite potential benefits from CEP use, only a fraction of TAVR procedures [16] is performed with any of the available CEP systems and data on large-scale consecutive CEP use is scarce.

Our center was among the first ones to use the Sentinel CEP device [13, 17] and has implemented CEP into its standard of care for performing TAVR since 2016. We analyzed in-hospital outcomes of our cohort of consecutive patients receiving CEP using the Sentinel device while undergoing TAVR.

## Methods

### Study population

This is a retrospective, single-center analysis of 2501 consecutive patients enrolled in the Ulm TAVR registry undergoing TAVR at the Ulm University Heart Center between January 2016 and December 2023. All patients provided written informed consent for data collection. The registry has been approved by the local ethics committee (Ulm University Ethics Committee, approval number: 283/21). This study and the Ulm TAVR registry comply with the standards set out in the Declaration of Helsinki. Sixty-nine patients who did not receive CEP during the enrollment period because of their inclusion in the PROTECTED TAVR trial [8] and randomization to the non-CEP arm were excluded from the current analysis. Furthermore, 7 patients (all of whom had received a CEP) suffering from annular rupture or requiring emergency conversion to open heart surgery were excluded. Apart from that, no further exclusion criteria existed.

All patients were evaluated by the local heart team before receiving TAVR. CEP use during TAVR procedures has been the standard of care at our heart center since 2016; thus, all patients are screened for CEP with the Sentinel CEP system.

### TAVR procedures

For procedural planning, patients underwent preprocedural 256 multislice contrast-enhanced computed tomography, which was evaluated with dedicated software (3mensio Structural Heart 9.1 software, Pie Medical Imaging B.V., Maastricht, The Netherlands). Preprocedural planning of TAVR procedures followed the local standardized protocol, which includes sizing of the aortic annulus, left-ventricular outflow tract, sinotubular junction, ascending aorta and assessment of the access route via the right or left groin in accordance with principles outlined in an expert consensus document [18]. Based on the measurements of the aortic annulus, the type and size of the TAVR prosthesis were chosen by the interventionalist according to the prosthesis manufacturer’s instructions for use. Annular calcification and LVOT calcification were graded according to Tops et al. [19] and Barbanti et al. [20], respectively. Furthermore, the anatomy (excessive tortuosity) and size of the brachiocephalic trunk and left common carotid artery as well as the anatomy of the aortic arch were visually evaluated to determine whether a patient was eligible for cerebral protection with the Sentinel device.

Procedures were performed under mild conscious sedation and local anesthesia of the groin using a transfemoral approach in a hybrid catheterization lab. Implantation was performed using fluoroscopy guidance without transesophageal echocardiography (TEE). All procedures were performed by an experienced team of interventionalists (board-certified cardiologists) in collaboration with cardiac surgeons.

The Sentinel CEP device was introduced via the right radial, ulnar or brachial artery using a 6 French sheath. The device consists of two filters with one filter placed in the brachiocephalic trunk and the other one in the left common carotid artery. Further details of the device have been described elsewhere [13]. According to our local standard, CEP was placed after the insertion of the TAVR delivery sheath, but before any device passes the aortic arch (via the transfemoral access). In patients with an indication for oral anticoagulation, such was discontinued ahead of the procedure. During the procedure, all patients received unfractionated heparin after the placement of the TAVR delivery sheath and activated clotting time (ACT) was targeted to 250–350 s including repeated measurements and additional heparin administration if the ACT target was not reached. Upon completion of the procedure, protamin was administered at a dose equivalent to half of the total heparin dose given.

### Study endpoints

The primary endpoint was ischemic stroke according to Valve Academic Research Consortium (VARC) 3-criteria (defined as acute onset of neurological signs or symptoms conforming to focal or multifocal central nervous system (CNS) territory with neuroimaging confirmation of CNS infarction, NeuroARC Type 1a [21]) within the index hospital stay after the TAVR procedure. Stroke severity was classified as non-disabling or disabling stroke, if the event led to death or discharge to another hospital.

Patients’ neurological status was assessed at the end of the procedure by the interventionalist and again upon return to the ward by the ward physician. The evaluation included vigilance, orientation, pronator drift, pupillary and oculomotor responses, visual deficits (neglect), muscle strength of the upper and lower extremities, and speech (dysarthria, aphasia) and sensation (light touch). Any newly developed neurological symptoms prompted immediate neurological consultation and neuroimaging. Intraprocedural stroke was defined as a stroke occurring before the patient left the catheterization laboratory.

Secondary endpoints were in-hospital minor and major vascular complications, bleeding, all-cause mortality, and in-hospital technical success [21].

Successful device placement was defined as both filters correctly deployed in the brachiocephalic trunk and left common carotid artery confirmed on fluoroscopy, respectively.

The primary analysis was conducted comparing patients with CEP to those who could not receive CEP for anatomical or technical reasons. In a secondary analysis, propensity score matching was performed to balance baseline differences between CEP/non-CEP patients to further quantify the impact of CEP use on the incidence of stroke. The CEP group included only patients with successful device placement.

### Statistical analysis

For statistical analysis, patients were grouped according to successful CEP deployment into a non-CEP and CEP patient group. Categorical variables are shown as absolute numbers and percentages and were compared using the chi-square or Fisher’s exact test, as appropriate. Continuous variables were assessed for normal distribution using histograms. Normally distributed variables were shown as mean ± standard deviation, whereas non-normally distributed variables were shown as median and interquartile range (IQR). Testing was conducted using the *t*-test and Mann–Whitney test, respectively.

In a secondary analysis, propensity score matching (PSM) was performed to account for baseline imbalances between patients undergoing TAVR without (non-CEP group) and with cerebral embolic protection (CEP group), targeting the average treatment effect on the controls (ATC). Covariates differing between the non-CEP and CEP groups in the unmatched cohort, identified based on both statistical and clinical relevance, were included in the propensity score model. Propensity scores were estimated using a multivariable logistic regression model incorporating the following variables: age, sex, NYHA class ≥ III, prior stroke or TIA, carotid artery disease, STS risk of mortality, predilation, arterial vessel disease, eGFR < 30 ml/min, and valve type (balloon-expandable, self-expandable, or mechanical). A 5:1 nearest-neighbor matching without replacement and a caliper width of 0.1 of the standard deviation of the logit of the propensity score were applied using the *MatchIt* package in R (RStudio version 2022.02.1). Although a 5:1 ratio was specified, the final number of matched pairs was lower because not every control observation (without CEP) had five sufficiently similar treated counterparts within the defined caliper range. Covariate balance between groups was evaluated using standardized mean differences (SMDs), with values < 0.1 indicating adequate balance. After matching, odds ratios for stroke and disabling stroke were derived from weighted logistic regression, with 95% confidence intervals and p-values calculated using cluster-robust (sandwich) standard errors based on the matching subclasses. All testing was performed two-sided and a *p*-value of < 0.05 indicated a significant result. Testing was performed using SPSS Version 28 (SPSS, IBM, Armonk, NY, USA). Propensity score matching was conducted using R (R Studio 2022.02.1, R Core Team (2022); R: A language and environment for statistical computing; and R Foundation for Statistical Computing, Vienna, Austria. URL https://www.R-project.org/).

## Results

### Baseline patient characteristics and reasons for not receiving CEP

**Fig. 1:**
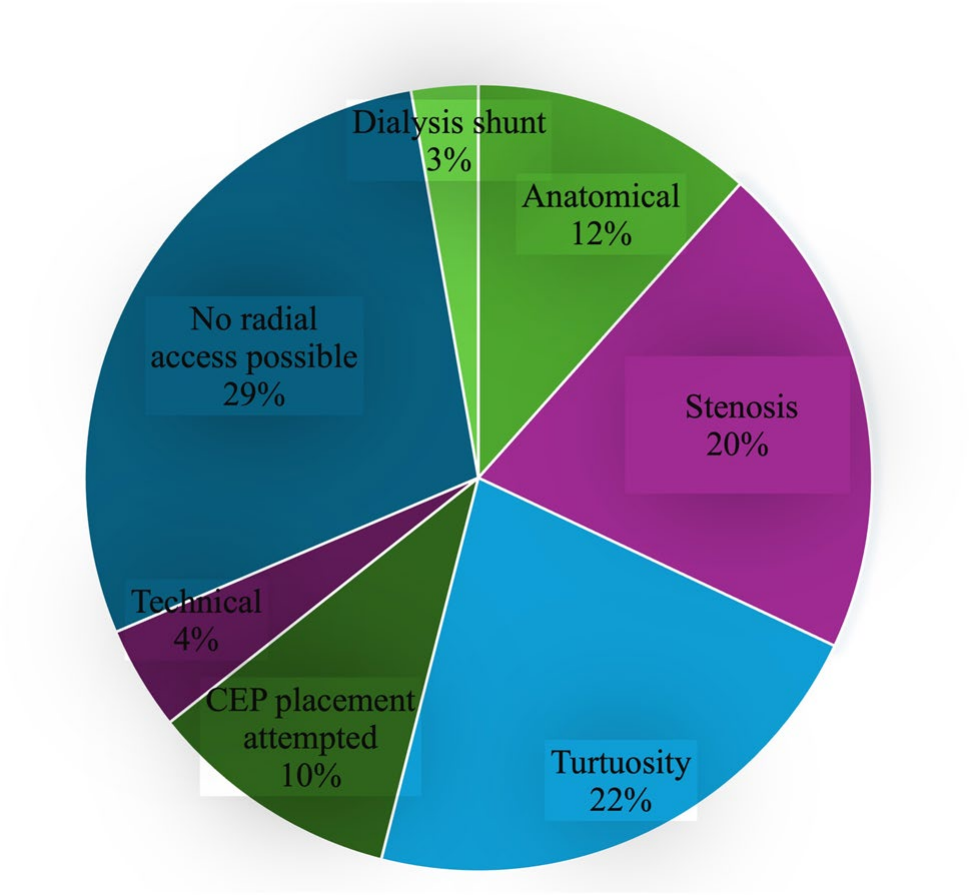
Pie chart depicting reasons for not using the Sentinel device in the study cohort. Successful placement of the Sentinel CEP system was achieved in 2173 patients (86.9%). Three hundred twenty-eight patients could not receive CEP with the Sentinel device. In case of “technical reasons,” vascular access was required for procedures other than CEP placement

In total, 2501 patients were included (see Table 1). Two thousand one hundred seventy-three patients received TAVR with the use of a CEP device, whereas 328 (13.1%) patients did not receive a CEP (non-CEP patients). No proper vessel access (28.7%), severe tortuosity (22.0%), and stenosis (brachiocephalic trunk, carotid artery; 20.4%) were the most frequent reasons for not receiving a CEP (see Fig. 1). Furthermore, in 34 (10.4%) patients, the device could not be placed successfully.

**Table 1:** Baseline patient characteristics

	Total *N* = 2501	No CEP *N* = 328	CEP *N* = 2173	*p*
Age, years	81.0 [77.0–85.0]	81.0 [77.0–85.0]	81.0 [77.0–84.0]	0.33
STS-Score Risk of Mortality, %	3.6 [2.2–6.0]	5.2 [3.3–7.9]	3.4 [2.2–5.9]	** < 0.01**
NYHA	3 [2, 3]	3 [3, 3]	3 [2, 3]	**0.02**
NYHA ≥ III, n	1867 (74.7)	259 (79.0)	1608 (74.0)	0.054
Female, *N* (%)	1186 (47.4)	177 (54.0)	1009 (46.4)	**0.01**
BMI, kg/m^2^	27.2 ± 4.8	27.1 ± 5.3	27.2 ± 4.8	0.77
Diabetes mellitus, *N* (%)	723 (28.9)	93 (28.4)	630 (29.0)	0.81
Coronary artery disease, *N* (%)	1548 (61.9)	208 (63.4)	1340 (61.7)	0.54
Prior cardiac surgery, *N* (%)	247 (9.9)	39 (11.9)	208 (9.6)	0.19
History of stroke/TIA, *N* (%)	307 (12.3)	53 (16.2)	254 (11.7)	**0.02**
Atrial fibrillation, *N* (%)	957 (38.3)	139 (42.4)	818 (37.6)	0.10
Arterial vessel disease, *N* (%)	1300 (52.0)	169 (51.5)	1131 (52.0)	0.86
Peripheral artery disease, *N* (%)	328 (13.1)	64 (19.5)	264 (12.1)	** < 0.01**
Carotid artery disease, *N* (%)	976 (39.0)	98 (29.9)	878 (40.4)	** < 0.01**
Carotid artery stenosis > 70%, *N* (%)	74 (3.0)	7 (2.1)	67 (3.1)	0.34
Dialysis, *N* (%)	48 (1.9)	10 (3.0)	38 (1.7)	0.11
Echocardiography				
LVEF, %	54.1 ± 14.5	53.2 ± 16.4	54.3 ± 14.2	0.18
LVEF ≤ 35%, *N* (%)	308 (12.3)	45 (13.7)	263 (12.1)	0.41
AV meanPG, mmHg	40.2 ± 16.0	39.1 ± 16.9	40.4 ± 15.8	0.17
AV maxPG, mmHg	66.2 ± 24.7	63.6 ± 26.6	66.6 ± 24.4	**0.04**
Laboratory data				
eGFR, ml/min	57.8 ± 21.8	55.0 ± 20.3	58.2 ± 22.0	**0.01**
eGFR < 30 ml/min, *N* (%)	227 (9.1)	37 (11.3)	190 (8.7)	0.14
Hemoglobin, g/dl	12.5 ± 2.1	12.4 ± 2.1	12.4 ± 2.1	0.55
Medication				
Any antiplatelet and/or anticoagulant, *N* (%)	1949 (77.9)	253 (77.1)	1696 (78.0)	0.71
ASS, *N* (%)	1173 (46.9)	143 (43.6)	1030 (47.4)	0.20
Dual antiplatelet therapy, *N* (%)	219 (8.8)	30 (9.1)	189 (8.7)	0.79
NOAC, *N* (%)	656 (26.2)	92 (28.0)	564 (26.0)	0.42
NOAC or VKA, *N* (%)	791 (31.6)	108 (32.9)	683 (31.4)	0.59

Values are shown as frequencies (*N*) and percentages (%), mean ± standard deviation (SD), or median and interquartile range (IQR). Bold values indicate significant *p*-values, *AF* atrial fibrillation, *ASS* acetylsalicylic acid, *AV* aortic valve, *BMI* body mass index, *CAD* coronary artery disease, *eGFR* estimated glomerular filtration rate, *Hb* hemoglobin level, *LVEF* left-ventricular ejection fraction, *max PG* maximum pressure gradient, *mPG* mean pressure gradient, *NOAC* novel oral anticoagulation, *NYHA* New York Heart Association, *STS* Society of Thoracic Surgeons, *TIA* transitory ischemic attack, *VKA* vitamin K antagonist

Regarding baseline characteristics, non-CEP patients had significantly higher Society of Thoracic Surgeons score for mortality (5.2 [interquartile range (IQR): 3.3–7.9] vs. 3.4% [IQR: 2.2–5.9], *p* < 0.01) and were more often female (54.0 vs. 46.4%, *p* = 0.01). Symptom burden in terms of New York Heart Association class (≥ NYHA III: 79.0 vs 74.0%, *p* = 0.054) tended to be higher in non-CEP patients. Comorbidities such as coronary artery disease (63.4 vs. 61.7%, *p* = 0.54) and prior cardiac surgery (11.9 vs. 9.6%, *p* = 0.19) were equally frequent in both groups, whereas a history of prior stroke (16.2 vs. 11.7%, *p* = 0.02) was more frequent in non-CEP patients. A comparable number of patients (13.7 vs. 12.1%, *p* = 0.41) had severely impaired left-ventricular ejection fraction (LVEF ≤ 35%).

Aortic valve (severe calcification: 71.3 vs. 73.5%, *p* = 0.4) and LVOT calcification (Barbanti 2/3: 27.7 vs. 27.6%, *p* = 0.96) were similar between both groups (see Table 2). Renal function was worse in non-CEP patients (eGFR: 55.0 ± 20.3 vs. 58.2 ± 22.0 ml/min, *p* = 0.01), and more patients tended to require permanent dialysis (3.0 vs. 1.7%, *p* = 0.11).

**Table 2:** Procedural details

	Total *N* = 2501	No CEP *N* = 328	CEP *N* = 2173	*p*
Annular size and valve calcification (preprocedural computed tomography)				
Aortic annulus area, mm^2^	476.2 ± 118.2	478.5 ± 206.5	475.8 ± 98.3	0.70
Small annular area < 430 mm^2^, *N* (%)	773 (30.9)	114 (34.8)	659 (30.3)	0.11
Aortic annulus perimeter, mm	78.4 ± 15.9	77.5 ± 10.2	78.5 ± 16.6	0.29
Severe aortic cusp calcification*, *N* (%)	1832 (73.3)	234 (71.3)	1598 (73.5)	0.40
LVOT calcification Barbanti 2/3, *N* (%)	691 (27.6)	91 (27.7)	600 (27.6)	0.96
Type of implanted prosthesis				
BEV, *N* (%)	1023 (40.9)	130 (39.6)	893 (41.1)	0.62
SEV, *N* (%)	1114 (44.5)	168 (51.2)	946 (43.5)	** < 0.01**
Lotus, *N* (%)	348 (13.9)	25 (7.6)	323 (14.9)	** < 0.01**
Other, *N* (%)	16 (0.6)	5 (1.5)	11 (0.5)	**0.048**
Procedural details				
Pre-dilation, *N* (%)	2058 (82.3)	240 (73.2)	1818 (83.7)	** < 0.01**
Post-dilation, *N* (%)	110 (4.4)	21 (6.4)	89 (4.1)	0.058
Procedure time, min	54.0 [45.0–65.0]	53.0 [43.0–63.25]	55.0 [46.0–66.0]	** < 0.01**
Procedural outcome				
Occlusion of coronary ostia, *N* (%)	5 (0.2)	1 (0.3)	4 (0.2)	0.51
Pericardial tamponade, *N* (%)	5 (0.2)	1 (0.3)	4 (0.2)	0.51
Hypotension requiring inotropes, *N* (%)	274 (11.0)	53 (16.2)	221 (10.2)	** < 0.01**
Aortic valve regurgitation ≥ II, *N* (%)	14 (0.6)	2 (0.6)	12 (0.6)	0.71
Arrhythmia, *N* (%)	298 (11.9)	39 (11.9)	259 (11.9)	0.99
Vascular complications, *N* (%)	111 (4.4)	18 (5.5)	93 (4.3)	0.32
Death, *N* (%)	3 (0.1)	0 (0.0)	3 (0.1)	1.0
Technical success, *N* (%)	2423 (96.9)	314 (95.7)	2109 (97.1)	0.20

Values are shown as frequencies (*N*) and percentages (%), mean ± standard deviation (SD), or median and interquartile range (IQR). Bold values indicate significant *p*-values. *BEV* balloon-expandable valve, *LVOT* left-ventricular outflow tract, *SEV* self-expandable valve

^*^Grades 3 and 4 according to Tops et al. [19]

Most patients were on either a single antiplatelet drug or on oral anticoagulation with similar rates in non-CEP and CEP patients (77.1 vs. 78.0%, *p* = 0.71). Moreover, the use of novel oral anticoagulants (28.0 vs 26.0%, *p* = 0.42) was similar in both groups.

### Procedural outcome

The majority of patients received either a balloon-expandable (BEV) (40.9%) or self-expandable (SEV) valve (44.5%), whereas only a minority of patients were implanted with the mechanically expandable Lotus valve (13.9%) (see also Table 2). Balloon predilation was performed significantly more often in CEP patients (73.2 vs. 83.7%, *p* < 0.01). The use of SEV was more frequent in non-CEP patients (51.2 vs. 43.5%, *p* < 0.01). The Boston Lotus valve was initially still in use in 2016 at our center; thus, its use was more frequent in CEP patients (7.6 vs. 14.9%, *p* < 0.01).

Procedure time was significantly longer in CEP patients (55.0 min [IQR: 46.0–66.0] vs. 53.0 min [43.0–63.3], *p* < 0.01).

Severe intraprocedural complications such as death (0 vs. 0.1%, *p* = 1.0), arrhythmia (11.9 vs. 11.9%, *p* = 0.99) and vascular access-site complications (5.5 vs. 4.3%, *p* = 0.32) were equally frequent in both groups. Non-CEP patients required inotropes during the TAVR procedure significantly more often (16.2 vs. 10.2%, *p* < 0.01).

### In-hospital stroke and disabling stroke

**Fig. 2:**
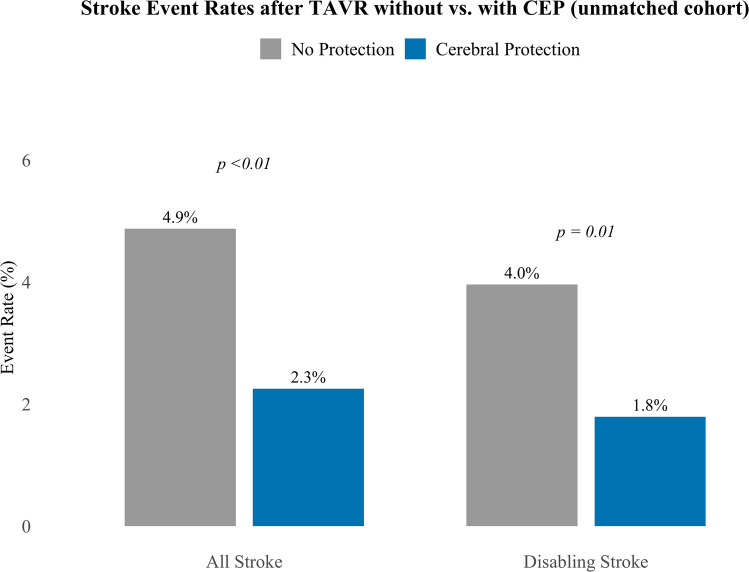
Stroke event rates after TAVR without vs. with cerebral embolic protection (unmatched cohort). Bar graphs show the incidence of all stroke and disabling stroke after transcatheter aortic valve replacement (TAVR) in patients without and with cerebral embolic protection (CEP) in the unmatched cohort. Percentages indicate observed event rates in each group. *p*-values were derived from chi-square tests

**Fig. 3:**
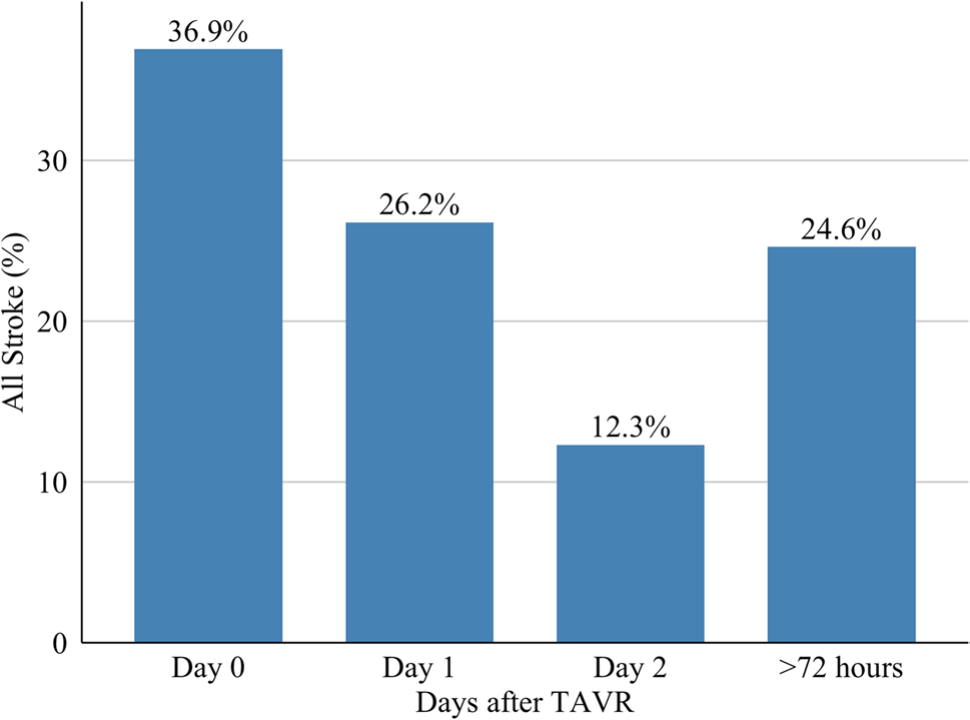
Bar graph depicting the timeline of strokes after TAVR. Most strokes occur within the first day of the procedure

2.6% of patients experienced stroke during their hospital stay. However, very few patients experienced intraprocedural stroke (0.2%). In-hospital disabling stroke occurred more often in non-CEP patients (4.0 vs. 1.8%, *p* = 0.01, see Fig. 2). Among those patients with an attempted but failed CEP placement, one patient experienced in-hospital stroke. The majority of stroke events occurred within the first 24 h after TAVR (36.9%) and 48 h after TAVR (26.2%; see also Fig. 3).

### In-hospital mortality and in-hospital complications

Technical success was high in the overall cohort (96.9%) but tended to be less frequent in non-CEP patients (95.7 vs. 97.1%, *p* = 0.2). Vascular (8.2 vs. 10.1%, *p* = 0.28) and major vascular complications (0.3 vs. 0.4%, *p* = 1.0) were equally frequent in non-CEP and CEP patients. Access-site complications were related to the TAVR delivery sheath, whereas none of the complications were related to the Sentinel device.

A similar number of patients required permanent pacemaker implantation (15.5 vs.13.1%, *p* = 0.23) due to conduction disturbances. In-hospital mortality was significantly higher in non-CEP patients (3.0 vs. 1.2%, *p* = 0.02).

### Outcomes after propensity score matching

Due to the caliper restriction, the final matched sample after 5:1 ATC PSM included 319 controls (9 unmatched patients) and 1339 treated cases, resulting in an effective mean matching ratio of approximately 4.2:1. Baseline characteristics were well balanced between groups, with standardized mean differences < 0.1 for all covariates, confirming adequate matching (see Supplemental Fig. 1). Supplemental Tables 1–3 depict baseline and procedural characteristics as well as in-hospital outcomes in the PSM cohort (Table 3).

**Table 3:** In-hospital outcome

	Total *N* = 2501	No CEP *N* = 328	CEP *N* = 2173	*p*-value
Permanent pacemaker implantation, *N* (%)	336 (13.4)	51 (15.5)	285 (13.1)	0.23
Vascular complication (hematoma, bleeding), *N* (%)	247 (9.9)	27 (8.2)	220 (10.1)	0.28
Major vascular complication, *N* (%)	10 (0.4)	1 (0.3)	9 (0.4)	1.0
Bleeding, *N* (%)	206 (8.2)	22 (6.7)	184 (8.5)	0.28
Major bleeding, *N* (%)	31 (1.2)	3 (0.9)	28 (1.3)	0.79
Acute kidney injury, *N* (%)	44 (1.8)	8 (2.4)	36 (1.7)	0.32
Mortality, *N* (%)	37 (1.5)	10 (3.0)	27 (1.2)	**0.02**
Stroke total, *N* (%)	65 (2.6)	16 (4.9)	49 (2.3)	** < 0.01**
Stroke intraprocedural, *N* (%)	6 (0.2)	0	6 (0.3)	1.0
Stroke postprocedural, *N* (%)	59 (2.4)	16 (4.9)	43 (2.0)	** < 0.01**
Disabling stroke, *N* (%)	52 (2.1)	13 (4.0)	39 (1.8)	**0.01**

Values are shown as frequencies (*N*) and percentages (%). Bold values indicate significant *p*-values

**Fig. 4:**
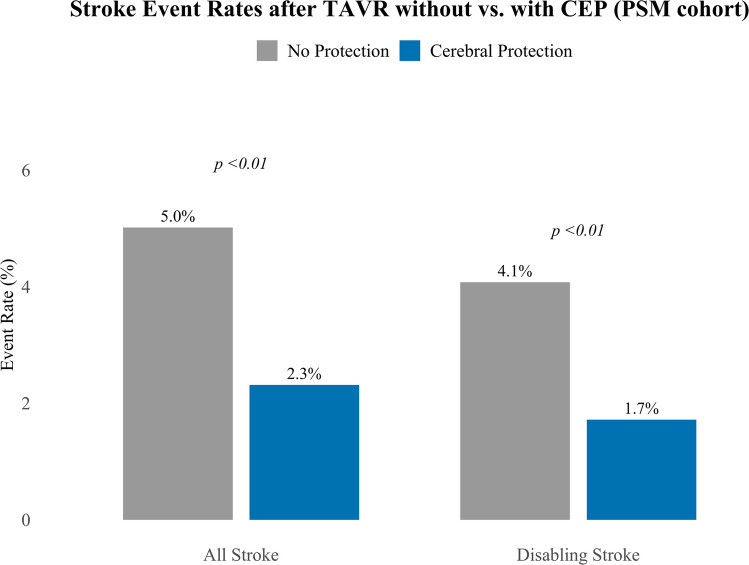
Stroke event rates after TAVR without vs. with cerebral embolic protection (propensity-score matched cohort). Bar graphs depict the incidence of all stroke and disabling stroke after TAVR comparing patients without and with cerebral embolic protection (CEP). Percentages indicate observed event rates in each group. *p*-values were derived from chi-square tests

**Fig. 5:**
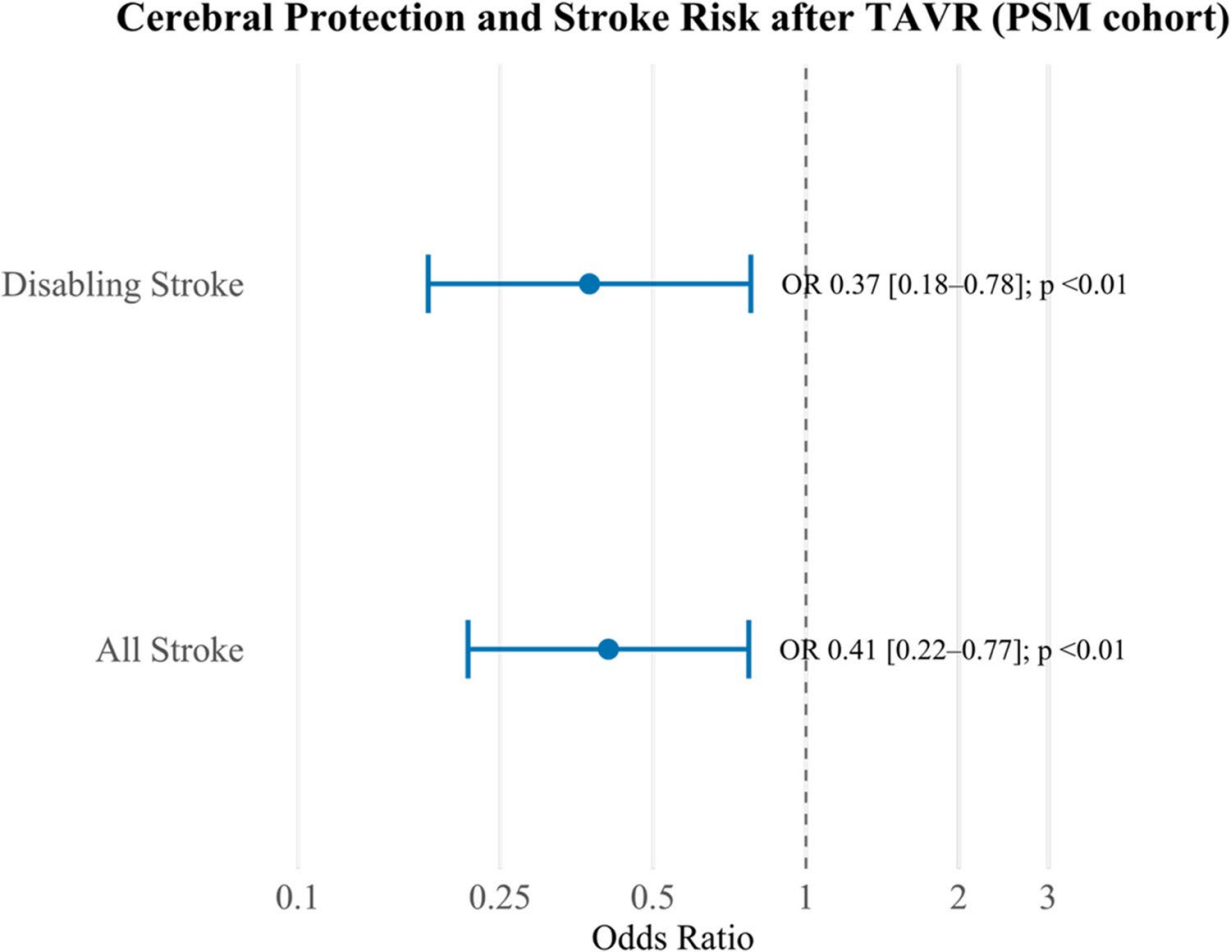
Forest plot showing the odds ratios (OR) with 95% confidence intervals (derived from weighted logistic regression) for all stroke and disabling stroke after transcatheter aortic valve replacement (TAVR) without versus with the use of CEP in the propensity score matched cohort. The dashed vertical line indicates an OR of 1.0. Values < 1.0 indicate a lower risk of stroke associated with the use of cerebral protection

In the matched cohort, stroke (5.0 vs. 2.3%, *p* < 0.01) as well as disabling stroke (4.1 vs. 1.7%, *p* < 0.01) occurred significantly more often in patients without CEP (see Fig. 4). This translated into a significantly lower risk of stroke with the use of cerebral protection during TAVR regarding all stroke (OR: 0.41, 95% CI 0.22–0.77; *p* < 0.01) as well as disabling stroke (OR: 0.37, 95% CI 0.18–0.78; *p* < 0.01; see Fig. 5) in the weighted logistic regression analysis.

## Discussion

CEP during TAVR aims to reduce the incidence of clinically evident early stroke caused by embolic debris and showed promising results by reducing the number and volume of cerebral lesions on MRI scans [9, 10] as well as by reducing the incidence of stroke in retrospective and propensity-score matched studies [13, 17]. Among the available CEP devices the Sentinel is the most frequently used system with the most data available in the literature [14]. CEP is increasingly critically viewed after the sobering results of the PROTECTED TAVR trial, which could neither establish nor rule out a clear benefit of CEP with the Sentinel system [8]. In an observational analysis of a large multicenter registry analyzing 18.725 TAVR procedures with 416 strokes, CEP use had no effect on the rate of all-stroke or disabling stroke [22]. Contrarily, the largest study to date including 414.649 patients found CEP use to be associated with a modest reduction in disabling strokes [23]

Our center has implemented CEP with the Sentinel system into its standard of care shortly after the Sentinel device was released into the market and we currently present the largest analysis of consecutive CEP use at a high-volume center. We compared CEP patients to those who could not receive CEP (with the Sentinel device) for anatomical or technical reasons and the main findings of our study can be summarized as follows:

- CEP with the Sentinel device can be performed in most patients (86.9%) in an all-comer population treated with TAVR.
- CEP use was not associated with an increase in vascular access site or other complications.
- Vessel stenosis and unsuitable anatomy (20.4 and 11.6%), severe tortuosity/kinking (22.0%) and lack of proper vascular access (28.7%) for introduction of a delivery sheath constitute the main reasons for not using CEP, whereas attempted but unsuccessful placement of the Sentinel device (10.4%) occured less often.
- Patients not receiving CEP had higher procedural risk according to the STS score and had a higher proportion of comorbidities, e.g. where sicker, and thus more prone to experience complications. Thus, the rate of in-hospital disabling stroke was higher in patients who could not receive CEP (4.0 vs. 1.8%, *p* = 0.01).
- After propensity score matching balancing baseline differences between groups, CEP use remained associated with a lower risk of all-stroke (OR: 0.41, 95%CI: 0.22–0.77, *p* < 0.01) as well as disabling stroke (OR: 0.37, 95%CI: 0.18–0.78, *p* < 0.01).

The highest level of evidence regarding CEP use originated from the randomized-controlled PROTECTED TAVR trial, which found a similar, but lower than expected overall rate of stroke, in patients with and without CEP [8]. The authors concluded that a potential benefit of CEP could not be ruled out due to the confidence intervals around this outcome (2.3 vs. 2.9%, 95% confidence interval (CI): − 1.7 to 0.5%). Moreover, a notable trend towards a reduction in disabling strokes (1.5 vs. 0.5%; 95% CI: − 1.5 to − 0.1) was observed [8]. In the preselected patient population enrolled in PROTECTED TAVR, CEP placement was successful in 94.4% and vascular complications at the access site were rare (0.1%) [8]. In line with the PROTECTED TAVR trial [8] as well as other studies investigating CEP use [12, 13, 24], we did neither observe an increase in vascular access-site complications with CEP.

Overall, in our all-comer population successful CEP use was high (86.9%) but expectedly less frequent compared to the more selected population presented in an RCT like the PROTECTED-TAVR trial (94.4%) [8]. The frequency of successful CEP placements is however slightly lower compared to the initial experience at our center with successful device placement in 91.8% [13] and to the overall success rate of 94.6% observed at another center with consecutive CEP use [24]. Although data on all-comer populations is scarce, the available literature suggests successful Sentinel deployment may be possible in up to 90% in all-comer populations [13, 24, 25], but those populations were usually smaller [13, 24, 25] than the current one. Notably, the number of patients with attempted but failed placement of the device was low (10.4% of non-CEP patients) in our cohort. Furthermore, in a relevant number of patients (28.7% of non-CEP patients) vascular access for the introduction of a delivery sheath could not be established.

The incidence of ischemic stroke (4.9 vs. 2.3%, *p* < 0.01) and more importantly disabling stroke (4.0 vs. 1.8%, *p* = 0.01) was significantly higher in patients not receiving CEP in our study. Higher interventional risk according to the STS score and a more pronounced comorbidity burden suggest that those patients represent an especially vulnerable subgroup of patients, which could profit from further measures to prevent stroke, including the use of (a suitable) CEP. Importantly, the results from our propensity score matched analysis strengthened this observation. By balancing key clinical and procedural characteristics between patients with and without CEP, PSM mitigated the baseline differences inherent to the nonrandomized design of the present study. In this 5:1 nearest-neighbor matching model targeting the average treatment effect on the controls (ATC), CEP use remained significantly associated with a lower risk of any stroke as well as disabling stroke.

Potentially, vulnerable subgroups of patients with inherently higher stroke risk might be underrepresented in trials and this might aid in explaining the mismatch of expected and observed strokes in trials such as PROTECTED-TAVR [8]. Furthermore, it has been suggested that patients perceived to be at very high risk for stroke might have been treated outside that trial [26]. Unfortunately, many of the larger pooled studies [16, 23] do not offer data on patients screened but deemed ineligible for Sentinel use, and usually only a fraction [16] of patients is protected with CEP in these larger analyses. Besides anatomical criteria (severe vessel tortuosity, vessel stenosis) rendering Sentinel use unfeasible, local expertise may influence the rate of successful Sentinel deployment. Thus, the available literature offers data only on patients in whom CEP was used, but little is known about those patients in whom it was not used. Our analysis aims to close this gap in knowledge and may aid in explaining why more dramatic reductions in stroke rates with CEP in contemporary studies have not been observed.

Consequently, we might not be able to protect some of the patients who would profit most from a CEP, e.g. with the Sentinel device. Due to the retrospective design of our study, it remains uncertain whether patients in whom the Sentinel could not be used, would have profited from a CEP device. However, our analysis shows that patients with higher a priori risk treated without CEP suffered from stroke more often.

Previously, numerous predictors of stroke following TAVR such as higher STS score [27], atrial fibrillation [16], and prior stroke [1, 7, 27] have been identified. Selective use of CEP [16] in high-risk patients, e.g., with prior stroke [23], might constitute an alternative, but raises ethical questions when considering that CEP might be beneficial, especially regarding disabling strokes. Selective CEP use, however, might reduce the number-needed-to-treat [23] to prevent strokes, thus offering a more cost-effective use of CEP. Our analysis similarly observed higher procedural risk according to the STS score for mortality as well as a higher rate of prior stroke/transitory ischemic attacks in patients not receiving CEP. This is in line with previous studies [16, 23] suggesting higher stroke risk in selective patient groups and emphasizes a potentially greater hazard for stroke patients who could not be protected with the Sentinel device in our analysis.

### Limitations

We presented results from a single-center, retrospective observational trial with all limitations inherent to such a study, thus all results must be considered hypothesis generating. A higher incidence of stroke in those patients not receiving CEP was observed. Given that the current study is not a randomized-controlled trial, causality should not be inferred from this. Moreover, non-CEP patients had inherently higher procedural risk according to the STS score for mortality. The CEP device was placed by board-certified (interventional) cardiologists with long-standing experience in vascular and coronary interventions as well as TAVR. Operator experience and institutional expertise derived from the years of CEP use may significantly impact the safety and rate of complications associated with the Sentinel device.

PSM was conducted to alleviate baseline differences between the non-CEP and CEP groups. While PSM mimics the effects usually achieved through controlled randomization it remains limited by unmeasured confounding. Nevertheless, standardized mean differences < 0.1 for all variables indicated excellent covariate balance, supporting the robustness of the matching procedure.

## Conclusion

In this retrospective observational study from a high-volume center using CEP as a standard of care during TAVR, CEP with the Sentinel device could be performed in the majority of patients without an increase in complications related to the device. Patients not receiving CEP for technical or anatomical reasons seemed to have a higher overall procedural risk and had a higher incidence of stroke and disabling stroke. After adjustment through propensity score matching, CEP remained associated with a lower risk of stroke, supporting its potential benefit in reducing periprocedural stroke during TAVR. Although speculative, patients who did not receive CEP due to technical or anatomical limitations might benefit from cerebral protection if these procedural constraints could be overcome in the future.

## Supplementary Information

Below is the link to the electronic supplementary material.

ESM 1 (DOCX 150 KB)
