# Supplementary material for: Cerebral embolic protection (CEP) during transcatheter aortic valve replacement (TAVR) is associated with a lower rate of stroke at a high-volume center
Source: Clin Res Cardiol. 2025 Nov 24;115(7):1218–29. doi: 10.1007/s00392-025-02802-5 (PMC13249724; doi:10.1007/s00392-025-02802-5)
Supplement: Supplementary file 1 — (DOCX 150 KB) [file 392_2025_2802_MOESM1_ESM.docx]

**Supplemental Figure 1: Covariate balance before and after propensity score matching (Love-Plot).**

The Love plot displays standardized mean differences (SMDs) for all covariates included in the propensity score model before (unadjusted) and after (adjusted) 5:1 nearest-neighbor matching targeting the average treatment effect on the controls (ATC). A standardized mean difference below 0.1 was considered indicative of adequate covariate balance between patients without and with cerebral embolic protection (CEP) after TAVR and was observed in all matched variables. Blue dots represent covariate balance after matching, and gray dots before matching. Horizontal dashed line denotes the predefined threshold for adequate balance (SMD = 0.1).


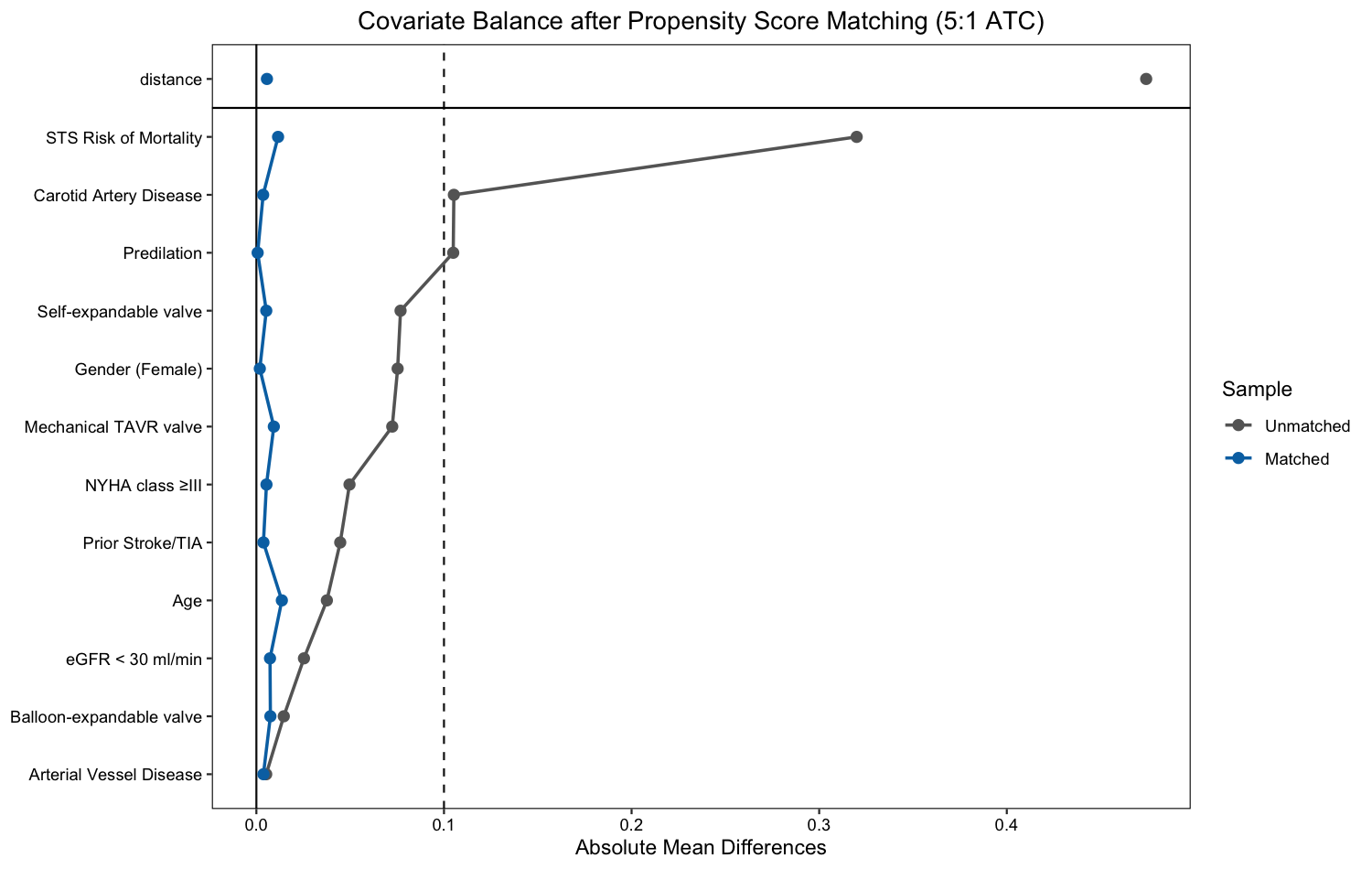


| **Supplemental Table 1. Baseline patient characteristics in the propensity score matched cohort** | | | | |
| --- | --- | --- | --- | --- |
|  | **Total**  **N=1658** | **No CEP**  **N=319** | **CEP**  **N=1339** | **p-value** |
| Age, years | 81.0 {77.0-85.0} | 82.0 {77.0-85.0} | 81.0 {77.0-85.0} | 0.47 |
| STS-Score Risk of Mortality, % | 4.0 {2.4-6.8} | 5.0 {3.1-7.6} | 3.8 {2.4-6.5} | **<0.01*** |
| NYHA | 3 {3-3} | 3 {3-3} | 3 {3-3} | 0.48 |
| NYHA ≥III, N (%) | 1293 (78.0) | 251 (78.7) | 1042 (77.8) | 0.74 |
| Female, N (%) | 863 (52.1) | 171 (53.6) | 692 (51.7) | 0.54 |
| BMI, kg/m^2^ | 27.2 ± 4.9 | 27.3 ± 5.2 | 27.2 ± 4.8 | 0.64 |
| Diabetes mellitus, N (%) | 494 (29.8) | 89 (27.9) | 405 (30.2) | 0.41 |
| Coronary artery disease, N (%) | 1028 (62.0) | 203 (63.6) | 825 (61.6) | 0.50 |
| Prior Cardiac Surgery, N (%) | 168 (10.1) | 37 (11.6) | 131 (9.8) | 0.33 |
| History of stroke/TIA, N (%) | 244 (14.7) | 51 (16.0) | 193 (14.4) | 0.48 |
| Atrial fibrillation, N (%) | 666 (40.2) | 131 (41.1) | 535 (40.0) | 0.72 |
| Arterial vessel disease, N (%) | 843 (50.8) | 164 (51.4) | 679 (50.7) | 0.82 |
| Peripheral artery disease, N (%) | 265 (16.0) | 61 (19.1) | 204 (15.2) | 0.09 |
| Carotid artery disease, N (%) | 551 (33.2) | 98 (30.7) | 453 (33.8) | 0.29 |
| Carotid artery stenosis >70%, N (%) | 46 (2.8) | 7 (2.2) | 39 (2.9) | 0.48 |
| Dialysis, N (%) | 40 (2.4) | 10 (3.1) | 30 (2.2) | 0.35 |
| **Echocardiography** | | | | |
| LVEF, % | 53.5 ± 15.1 | 53.2 ± 16.4 | 53.6 ± 14.8 | 0.67 |
| LVEF ≤35 %, N (%) | 221 (13.3) | 44 (13.8) | 177 (13.2) | 0.79 |
| meanPG, mmHg | 39.5 ± 16.6 | 39.1 ± 17.0 | 39.5 ± 16.5 | 0.70 |
| maxPG, mmHg | 65.2 ± 25.5 | 63.7 ± 26.7 | 65.5 ± 25.3 | 0.27 |
| **Laboratory data** | | | | |
| GFR, ml/min | 56.5 ± 21.5 | 55.0 ± 20.4 | 56.8 ± 21.7 | 0.19 |
| GFR <30ml/min, N (%) | 168 (10.1) | 36 (11.3) | 132 (9.9) | 0.45 |
| Hemoglobin, g/dl | 12.3 ± 2.1 | 12.3 ± 2.1 | 12.3 ± 2.1 | 0.89 |
| **Medication** | | | | |
| Any antiplatelet and/or anticoagulant, N (%) | 1298 (78.3) | 250 (78.4) | 1048 (78.3) | 0.97 |
| ASS, n | 772 (46.6) | 143 (44.8) | 629 (47.0) | 0.49 |
| Dual antiplatelet therapy, N (%) | 134 (8.1) | 30 (9.4) | 104 (7.8) | 0.34 |
| NOAC, N (%) | 454 (27.4) | 89 (27.9) | 365 (27.3) | 0.82 |
| NOAC or VKA, N (%) | 537 (32.4) | 105 (32.9) | 432 (32.3) | 0.82 |
| 5:1 ATC (average treatment effect on controls) propensity score matching (PSM) the cohort included 1339 CEP and 319 non-CEP patients (9 unmatched patients in the non-CEP group). Due to the caliper restriction, the final matched sample included 319 controls and 1339 treated cases, resulting in an effective mean matching ratio of approximately 4.2:1.  Values are shown as frequencies (N) and percentages (%), mean ± standard deviation (SD) or median and interquartile range (IQR). Bold values indicate significant p-values.  *After PSM STS score for mortality remained significantly different, however, the covariate balance confirmed adequate matching with an absolute mean difference <0.1 for the STS score after PSM.  Abbreviations: AF = atrial fibrillation, ASS = acetylic salcyclic acid, AV = aortic valve, BMI = body mass index, CAD = coronary artery disease, eGFR = estimated glomerular filtration rate, Hb = hemoglobin level, LVEF = left-ventricular ejection fraction, max PG = maximum pressure gradient, mPG= mean pressure gradient, NOAC = novel oral anticoagulation, NYHA = New York Heart Association, STS = Society of Thoracic Surgeons, TIA = transitory ischemic attack, VKA = vitamin K antagonist | | | | |

| **Table 2. Procedural details in the propensity score matched cohort** | | | | |
| --- | --- | --- | --- | --- |
|  | **Total**  **N=1658** | **No CEP**  **N=319** | **CEP**  **N=1339** | **p-value** |
| **Annular size and valve calcification (preprocedural computed tomography)** | | | | |
| Aortic annulus area, mm^2^ | 471.0 ± 129.4 | 479.6 ± 208.6 | 469.0 ± 101.8 | 0.19 |
| Small annular area  <430 mm^2^, N (%) | 569 (34.3) | 110 (34.5) | 459 (34.3) | 0.95 |
| Aortic annulus perimeter, mm | 78.0 ± 18.4 | 77.6 ± 10.2 | 78.1 ± 19.9 | 0.65 |
| Severe aortic cusp calcification, N (%) | 1157 (69.8) | 229 (71.8) | 928 (69.3) | 0.39 |
| LVOT calcification Barbanti 2/3, N (%) | 459 (27.7) | 88 (27.6) | 371 (27.7) | 0.97 |
| **Type of implanted prosthesis** | | | | |
| BEV, N (%) | 660 (39.8) | 127 (39.8) | 533 (39.8) | 1.0 |
| SEV, N (%) | 824 (49.7) | 165 (51.7) | 659 (49.2) | 0.42 |
| Mechanical, N (%) | 162 (9.8) | 25 (7.8) | 137 (10.2) | 0.20 |
| Other, N (%) | 12 (0.7) | 2 (0.6) | 10 (0.7) | 1.0 |
| **Procedural details** | | | | |
| Pre-dilation, N (%) | 1271 (76.7) | 234 (73.4) | 1037 (77.4) | 0.12 |
| Post-dilation, N (%) | 82 (4.9) | 19 (6.0) | 63 (4.7) | 0.35 |
|  |  |  |  |  |
| Procedure time, min | 53.0 {45.0-64.0} | 52.0 {42.0-63.0} | 53.0 {45.0-65.0} | 0.13 |
| **Procedural outcome** | | | | |
| Occlusion of coronary ostia, N (%) | 2 (0.1) | 1 (0.3) | 1 (0.1) | 0.35 |
| Pericardial tamponade, N (%) | 3 (0.2) | 1 (0.3) | 2 (0.1) | 0.47 |
| Hypotension requiring inotropes, N (%) | 198 (11.9) | 50 (15.7) | 148 (11.1) | **0.02** |
| Aortic valve regurgitation ≥ II, N (%) | 7 (0.4) | 2 (0.6) | 5 (0.4) | 0.63 |
| Arrhythmia, n | 193 (11.6) | 38 (11.9) | 155 (11.6) | 0.87 |
| Vascular complications, N (%) | 78 (4.7) | 18 (5.6) | 60 (4.5) | 0.38 |
| Death, N (%) | 1 (0.1) | 0 (0.0) | 1 (0.1) | 1.0 |
| Technical success, N (%) | 1610 (97.1) | 305 (95.6) | 1305 (97.5) | 0.08 |
| Values are shown as frequencies (N) and percentages (%), mean ± standard deviation (SD) or median and interquartile range (IQR). Bold values indicate significant p-values.  *grade 3 and 4 according to Tops et al. [19] | | | | |

| **Supplemental Table 3. In-hospital outcome in the propensity score matched cohort** | | | | |
| --- | --- | --- | --- | --- |
|  | **Total**  **N=1658** | **No CEP**  **N=319** | **CEP**  **N=1339** | **p-value** |
| Permanent pacemaker  implantation, N (%) | 212 (12.8) | 51 (16.0) | 161 (12.0) | 0.057 |
| Vascular complications  (hematoma, bleeding), N (%) | 168 (10.1) | 27 (8.5) | 141 (10.5) | 0.27 |
| Major vascular complication, N (%) | 13 (0.8) | 2 (0.6) | 11 (0.8) | 1.0 |
| Bleeding, N (%) | 132 (8.0) | 22 (6.9) | 110 (8.2) | 0.43 |
| Major bleeding, N (%) | 18 (1.1) | 3 (0.9) | 15 (1.1) | 1.0 |
| Acute kidney injury, N (%) | 33 (2.0) | 8 (2.5) | 25 (1.9) | 0.46 |
| Mortality, N (%) | 28 (1.7) | 10 (3.1) | 18 (1.3) | **0.03** |
| Stroke total, N (%) | 47 (2.8) | 16 (5.0) | 31 (2.3) | **<0.01** |
| Stroke intraprocedural, N (%) | 5 (0.3) | 0 (0.0) | 5 (0.4) | 0.59 |
| Stroke postprocedural, N (%) | 42 (2.5) | 16 (5.0) | 26 (1.9) | **<0.01** |
| Disabling stroke, N (%) | 36 (2.2) | 13 (4.1) | 23 (1.7) | **<0.01** |
| Values are shown as frequencies (N) and percentages (%). Bold values indicate significant p-values. | | | | |
